# Association between exposure to outdoor artificial light at night and the risk of preterm birth

**DOI:** 10.3389/fpubh.2023.1280790

**Published:** 2023-12-14

**Authors:** Qi Sun, Yang Yang, Jing Liu, Fang Ye, Yuanmei Chen, Die Liu, Qi Zhang

**Affiliations:** ^1^National Center for Respiratory Medicine, State Key Laboratory of Respiratory Health and Multimorbidity, National Clinical Research Center for Respiratory Diseases, Institute of Respiratory Medicine, Chinese Academy of Medical Sciences, Department of Pediatrics, China-Japan Friendship Hospital, Beijing, China; ^2^Graduate School of Peking Union Medical College, Chinese Academy of Medical Sciences, Beijing, China

**Keywords:** preterm birth, artificial light at night, pregnancy, risk factors, environmental exposure

## Abstract

**Background:**

This study aimed to investigate the association between outdoor artificial light at night (ALAN) exposure during pregnancy and the risk of preterm birth (PTB).

**Methods:**

A retrospective case–control study was conducted, and data were collected from pregnant women residing in Beijing, China. The level of ALAN exposure during pregnancy was estimated using remote sensing satellite data. Propensity score matching was utilized to match the control group. Logistic and multivariate linear regression were used to analyze the association between ALAN and the risk of PTB. The odds ratio (OR) and partial regression coefficient (β) with 95% confidence interval (CI) were utilized to assess the association.

**Results:**

A total of 2,850 pregnant women were enrolled in this study. ALAN (nW/cm^2^/sr) exposure was higher in the PTB group than in the control group during first trimester (mean ± standard deviation: 25.30 ± 17.91 vs. 17.56 ± 14.74, *p* < 0.001) and second trimester (27.07 ± 18.10 vs. 21.93 ± 16.08, *p* < 0.001). A negative association was found between ALAN exposure and gestation day in the first (β = −0.151, 95%CI: −0.217 to −0.085, *p* < 0.001) and second trimesters (β = −0.077, 95%CI: −0.139 to −0.015, *p* = 0.015). ALAN was identified as a risk factor for PTB during the first trimester (OR = 1.032, 95%CI: 1.025–1.040, *p* < 0.001) and the second trimester (OR = 1.018, 95%CI: 1.011–1.025, *p* < 0.001), while no significant association was observed in the third trimester.

**Conclusion:**

Our study suggesting that exposure to outdoor ALAN, especially during first and second trimester, was associated with the risk of PTB. These findings highlight the potential impact of ALAN on pregnancy health and offer new insights into the risk of PTB.

## Introduction

Exposure to various outdoor artificial light at night (ALAN) is a pervasive environmental risk factor in modern society (1). In recent decades, urbanization and changes in modern lifestyles have led to an increasing exposure of individuals to ALAN in their daily lives (2). While ALAN provides safety and convenience, it also brings a range of potential health issues (3).

Until recent years, researchers had shown limited attention to the issue of global light pollution, despite over 80% of the global population being exposed to nighttime light pollution (4). Studies have shown that ALAN can disrupt the circadian rhythms of humans and other organisms, which regulate numerous physiological processes and behavioral activities (5–7). Exposure to ALAN may suppress the secretion of melatonin, a hormone crucial for sleep regulation and other physiological functions (8). Additionally, ALAN may affect the functionality of other endocrine systems, such as the secretion of adrenocorticotropic hormones and insulin regulation (9).

Using satellite remote sensing data, researchers have observed associations between ALAN and diseases such as obesity (10), atherosclerosis (11), sleep disorders (12), and cancer (13). However, limited evidence exists regarding the impact of outdoor ALAN on pregnancy health. Pregnant women, as a unique population, play a critical role in the health and development of both mother and child during pregnancy. During pregnancy, pregnant women undergo various physiological changes (14–16). These physiological changes can render pregnant women more susceptible to environmental factors (17). Current research has found that exposure to ALAN may negatively affect the sleep quality, endocrine system, and circadian rhythms of pregnant women, potentially influencing pregnancy outcomes (18–20). Nonetheless, the association between ALAN exposure during pregnancy and preterm birth (PTB) remains uncertain.

In China, PTB was a significant public health problem, primarily attributed to behavioral and environmental risk factors (21, 22). With rapid urbanization and economic development, ALAN in Chinese cities has significantly increased (23). Individuals living in urban areas were more likely to transition from the natural 24-h light–dark cycle to patterns involving round-the-clock work, late-night activities, and exposure to ALAN. Therefore, there was a need to assess the potential risks of PTB associated with ALAN exposure and formulate effective prevention strategies.

To fill the existing knowledge gaps regarding the association between outdoor ALAN exposure during pregnancy and PTB, we conducted a retrospective case–control study in Beijing, China. This investigation seeks to shed light on the crucial aspects of ALAN’s impact on pregnancy outcomes.

## Materials and methods

### Study population

**Figure 1:**
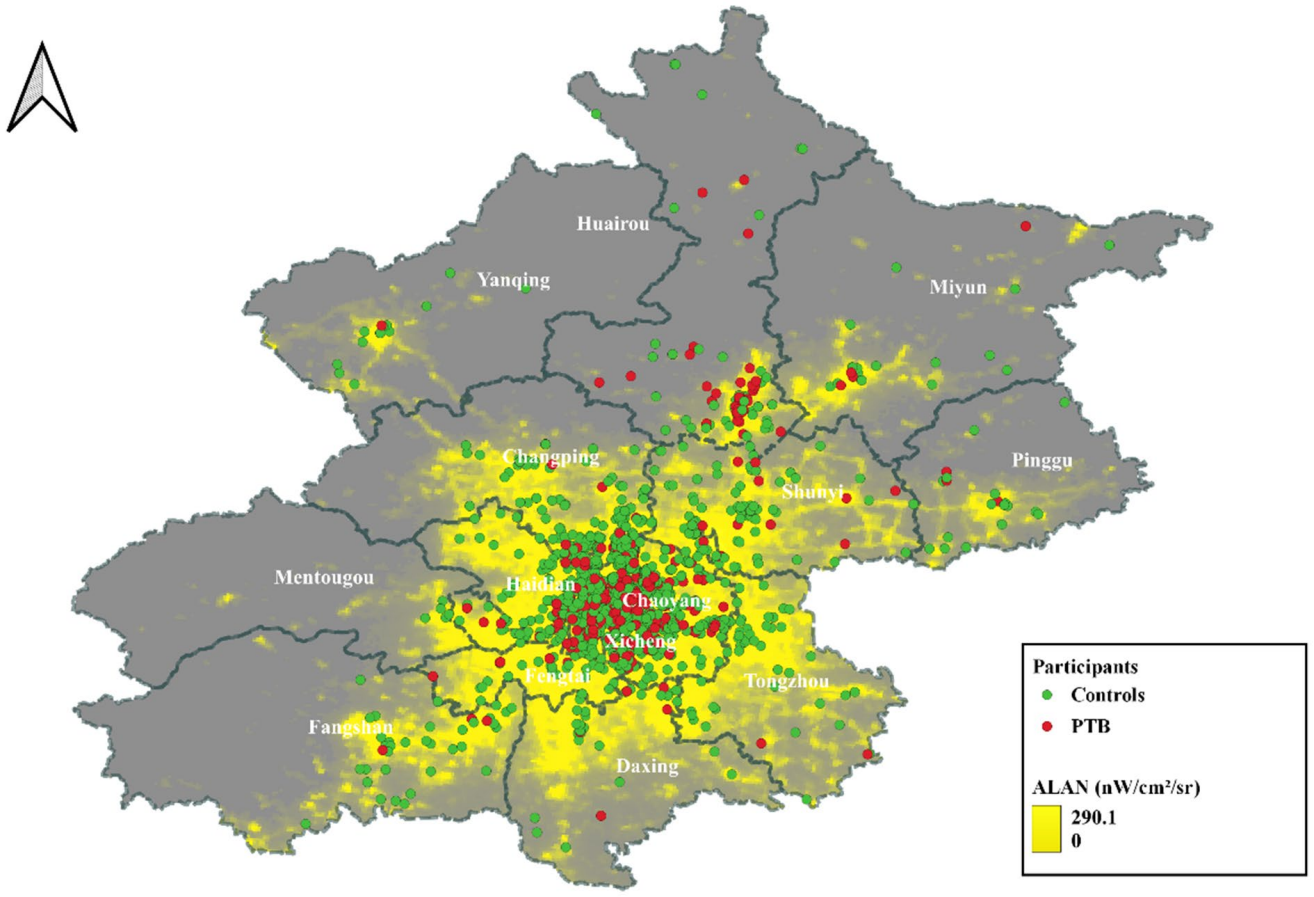
Geographical distribution of participants in Beijing. ALAN, Artificial light at night; PTB, Preterm birth; Red dots represent PTBs, and green dots represent term birth of pregnant women.

**Figure 2:**
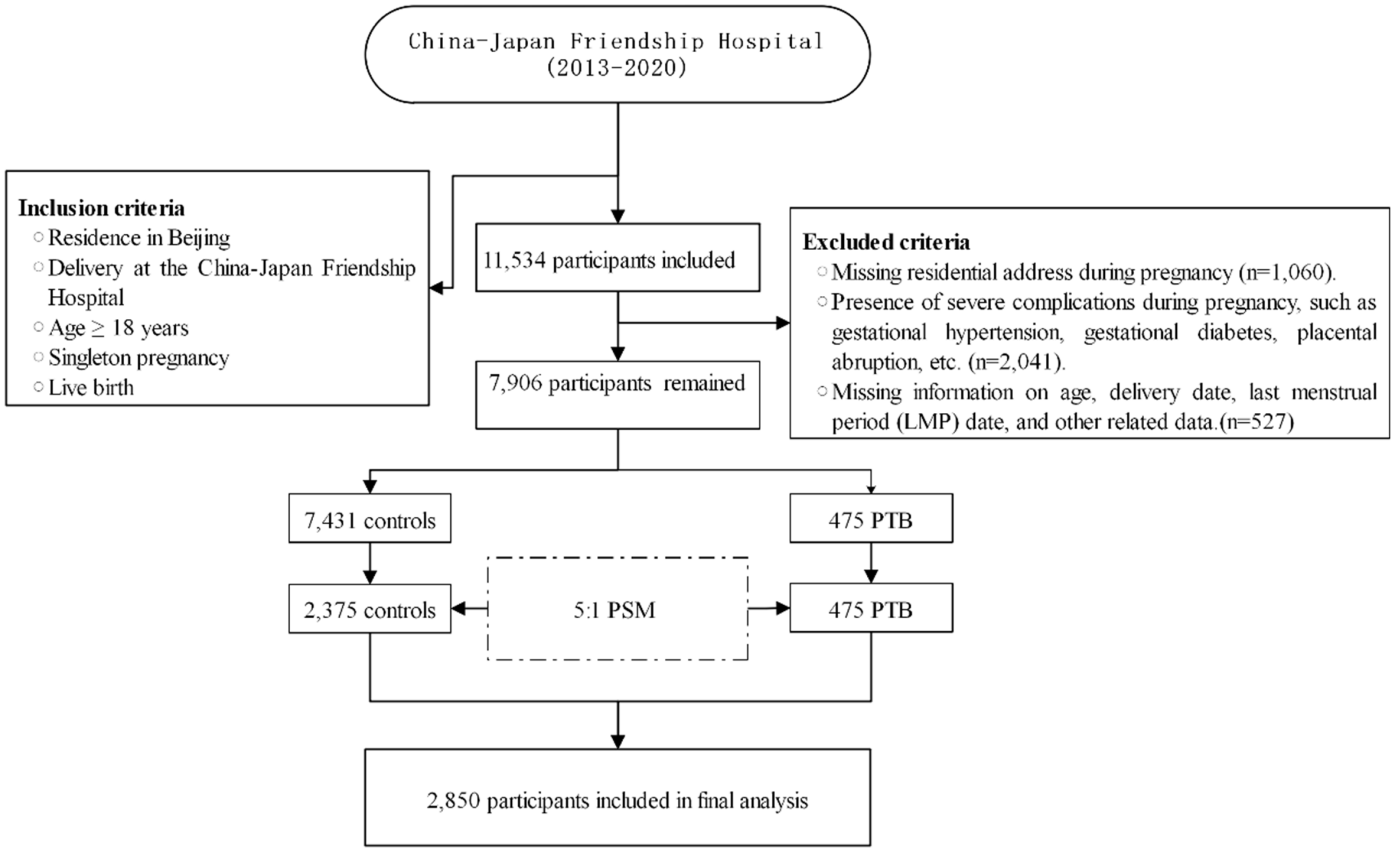
Flowchart of the study. LMP, Last menstrual period; PTB, Preterm birth; PSM, Propensity score matching.

This retrospective case–control study was conducted at the China-Japan Friendship Hospital. The geographic distribution of study subjects was depicted in Figure 1. Participants were selected based on specific inclusion criteria, which included the following: (1) Residence in Beijing; (2) Delivery at the China-Japan Friendship Hospital; (3) Maternal age ≥ 18 years; (4) Singleton pregnancies; and (5) Live births. Exclusion criteria comprised the following: (1) Missing residential address during pregnancy; (2) Severe pregnancy complications (e.g., gestational hypertension, gestational diabetes, and placental abruption); and (3) Incomplete essential information (e.g., age, delivery date, and last menstrual period date). Propensity score matching with a 1:5 ratio was used to select controls based on age, ethnicity, parity, and gravidity. The final study comprised 2,850 subjects, and its workflow was presented in Figure 2.

This retrospective case–control study design precluded the acquisition of informed consent from the participants. However, this approach was in accordance with the ethical approval obtained from the Ethics Committee of the China-Japan Friendship Hospital (No. 2023-KY-137), which acknowledged the impracticality of obtaining informed consent in a retrospective study.

### Assessment of outdoor ALAN

To date, two types of ALAN satellite data were utilized in human health research, including the Operational Linescan System of Defense Meteorological Satellite Program (OLS-DMSP) and the Suomi National Polar-Orbiting Partnership Visible Infrared Imaging Radiometer Suite (NPP-VIIRS) (24, 25). DMSP-OLS was launched in 1992, with a spatial resolution of 1 km × 1 km for its data products (26). Compared to OLS-DMSP, NPP-VIIRS provides higher spatial resolution, improved temporal resolution, expanded spectral range, and advanced calibration and correction (27). Therefore, in this study, we utilized monthly NPP-VIIRS as the source of ALAN data. NPP-VIIRS, beginning in April 2012, captures data within the wavelength range of 500–900 nm, with a spatial resolution of 500 m × 500 m at the Equator (28). The unit of measurement is nanowatts per square centimeter per steradian (nW/cm^2^/sr), which quantifies the radiative intensity per unit area, considering the solid angle spanning all directions. Monthly NPP-VIIRS nighttime light data for the period from 2013 to 2020 were obtained from the Earth Observation Group,^1^ in the GeoTIFF file format.

### Outcomes and covariates

The patients’ last menstrual date and delivery date were collected, and based on these dates, the gestational age of the pregnant women was calculated. If the gestational age was ≤37 weeks, it was defined as PTB (29). This study simultaneously collected data on fetal sex and birth weight. Additionally, the following covariates were also collected: maternal ethnicity (Han, non-Han), age (years), parity (primiparous, multiparous), gravidity (nulliparous, one or more), and gestational age (weeks).

### Other environmental variables

Considering the role of environmental factors in PTB, we incorporated ambient inhalable particulate matter (PM_10_) and ambient fine particulate matter (PM_2.5_), along with green space, as environmental covariates. The PM_2.5_ and PM_10_ data were obtained from China High Air Pollutants (CHAP). PM_2.5_ and PM_10_ data were derived using a spatio-temporal extreme random tree model, which incorporated model data to fill in the spatial gaps of the Moderate Resolution Imaging Spectroradiometer Multi-Angle Implementation of Atmospheric Correction Aerosol Optical Depth satellite product. This approach combined ground observations, atmospheric reanalysis, emission inventories, and other big data sources to generate seamless nationwide surface PM_2.5_ and PM_10_ data from 2000 to 2021. The 10-fold cross-validation coefficient of determination (*R*^2^) for PM_2.5_ data was 0.92, with a root mean square error (RMSE) of 10.76 μg/m^3^ (30). For PM_10_ data, the 10-fold cross-validation *R*^2^ was 0.9, with an RMSE of 21.12 μg/m^3^ (31). The normalized difference vegetation index (NDVI) was used as a proxy for residential greenness. NDVI was a widely used indicator in environmental research that quantifies the density and health of vegetation in each area (32). The index ranges from 0 to 1, where higher NDVI values indicate denser and healthier vegetation, while lower values indicate sparse or stressed vegetation (33). In our study, NDVI was estimated based on 16-day composite images derived from the Terra Moderate Resolution Imaging Spectroradiometer satellite of NASA.^2^ After acquiring annual data on PM_2.5_, PM_10_, and NDVI, we performed a matching of pregnant women’s residential weights during the gestation period and computed the yearly gestational environmental pollution exposure.

### Exposure time window

**Figure 3:**
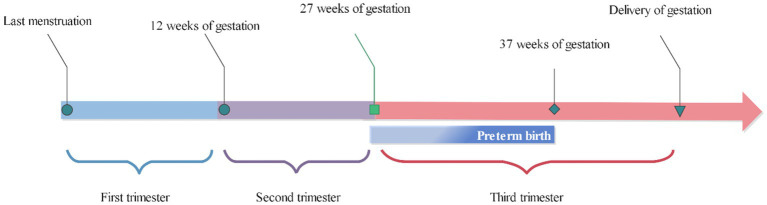
Definition of the exposure window.

The participants’ residential addresses were geocoded using Baidu Maps.^3^ Subsequently, we proceeded to estimate the average exposures during first, second, and third trimester to investigate potential heterogeneity in the association between various exposure windows of ALAN and PTB. The exposure windows encompassed the first, second, and third trimesters of pregnancy, corresponding to 3, 6, and up to delivery after the last menstrual period, respectively. The definition of exposure windows was presented in Figure 3.

### Statistical analysis

Continuous variables following a normal distribution were expressed as mean ± standard deviation, while categorical variables were presented as counts (percentages). Differences in continuous variables between groups were compared using the t-test or Wilcoxon test. Differences in categorical variables between groups were compared using the chi-square test or Fisher’s test.

A multivariable linear regression model was fitted to estimate the association between outdoor ALAN and gestational days, with these indicators being modeled as continuous variables. We initially established an unadjusted crude model without considering any potential confounding factors. Subsequently, we adjusted for potential confounders. Finally, we further controlled for PM_2.5_, PM_10_, and NDVI based on addressing potential confounding sources. The association between ALAN and PTB was analyzed using a binary logistic regression model, and the odds ratio (OR) along with its 95% confidence interval (CI) was reported. Furthermore, we performed stratified analysis by infant sex to examine potential effect modification and to assess the interaction between ALAN and infant sex. All statistical analyses were performed using R (version 4.1.0, available from: https://www.r-project.org/).

### Sensitivity analysis

Several sensitivity analyses were conducted in this study: (1) ALAN was categorized into five categories based on percentiles and included in all analysis to assess the influence of a 20% increase in ALAN on the outcomes (Supplementary Tables S1–S4). (2) Evaluation of participants of non-Han ethnicity to assess the potential impact of ethic (Supplementary Table S5). (3) Similar analysis was conducted in the primiparous population to assess potential differences attributed to multiparity (Supplementary Table S6).

## Results

### Characteristics of the study population

A total of 2,850 pregnant women were included in this study, with no significant differences observed between the two groups in terms of ethnicity, age, gravidity, and parity. The proportion of male newborns was higher in the preterm group compared to the control group (PTB group: 57.26%, control group: 50.69%, *p* = 0.010). Newborns in the PTB group had shorter length (PTB group: 45.89 ± 4.41 cm, control group: 51.06 ± 1.85 cm, *p* < 0.001) and lower birth weight (PTB group: 2386.57 ± 614.07 g, control group: 3374.72 ± 412.38 g, *p* < 0.001) compared to the control group. Gestational age in PTB group lower than that in control group (PTB group: 243.08 ± 17.11 days, control group: 278.35 ± 25.87 days, *p* < 0.001). All participant characteristics were presented in Table 1.

**Table 1:** Characteristics of pregnant women and newborns.

Variables		Control group (*n* = 2,375)	PTB group (*n* = 475)	*p*
Han Chinese (%)	No	107 (4.51)	22 (4.63)	1.000
	Yes	2,268 (95.49)	453 (95.37)	
Age (years)		31.44 ± 3.71	31.66 ± 4.14	0.262
Multipara (%)	No	1720 (72.42)	342 (72.00)	0.896
	Yes	655 (27.58)	133 (28.00)	
Gravidity (times)	0	1,380 (58.11)	272 (57.26)	0.516
	1	566 (23.83)	107 (22.53)	
	≥ 2	429 (18.06)	96 (20.21)	
Neonatal sex (%)	Male	1,204 (50.69)	272 (57.26)	0.010
	Female	1,171 (49.31)	203 (42.74)	
Neonatal length (cm)		51.06 ± 1.85	45.89 ± 4.41	<0.001
Birth weight (g)		3374.72 ± 412.38	2386.57 ± 614.07	<0.001
Days of pregnancy (days)		278.35 ± 25.87	243.08 ± 17.11	<0.001

### Distribution of environmental factors in different trimesters

There were no statistically significant differences in ambient PM_2.5_ and PM_10_ levels between the two groups across trimesters, while the vegetation index was higher in the control group compared to the PTB group (*p* = 0.001). ALAN (nW/cm^2^/sr) was significantly higher in the PTB group compared to the control group during the first (PTB group: 25.30 ± 17.91, control group: 17.56 ± 14.74, *p* < 0.001) and second trimesters (PTB group: 27.07 ± 18.10, control group: 21.93 ± 16.08, *p* < 0.001, respectively; Table 2). The distribution of ALAN between the two groups was depicted in Supplementary Figure S1. However, there were no statistically significant differences in ALAN exposure during the third trimester between the two groups.

**Table 2:** Differences in outdoor ALAN between the PTB and control groups.

Variables	Control group	PTB group	*p*
PM_10_ (μg/m^3^)	102.29 ± 20.74	103.01 ± 21.05	0.491
PM_25_ (μg/m^3^)	64.02 ± 17.01	65.17 ± 17.65	0.183
NDVI	0.33 ± 0.07	0.32 ± 0.07	0.001
ALAN _T1_ (nW/cm^2^/sr)	17.56 ± 14.74	25.30 ± 17.91	<0.001
ALAN _T2_ (nW/cm^2^/sr)	21.93 ± 16.08	27.07 ± 18.10	<0.001
ALAN _T3_ (nW/cm^2^/sr)	24.85 ± 16.61	26.76 ± 19.67	0.057

ALAN, Artificial light at night; NDVI, Normalized difference vegetation index; T1, First trimester; T2, Second; T3, Third trimester.

### Association of outdoor ALAN exposure in different trimesters with gestation day

Table 3 presents the results of the multiple linear regression model examining the association between outdoor ALAN exposure and gestation day. In the crude model, a significant negative association was observed between ALAN exposure and gestation day in the first trimester (T1: β = −0.151, 95%CI: −0.217 to −0.085, *p* < 0.001) and second trimester (T2: β = −0.077, 95%CI: −0.139 to −0.015, *p* = 0.015), whereas no significant association was found in the third trimester. After adjusting for age, ethnicity, gravidity, and parity, similar results were obtained. In the fully adjusted model, which additionally accounted for NDVI, PM_2.5_, and PM_10_, the associations between ALAN exposure and gestation day in the first trimester (T1: β = −0.156, 95%CI: −0.201 to −0.111, *p* < 0.001) and second trimester (T2: β = −0.076, 95%CI: −0.120 to −0.033, *p* = 0.001) remained consistent.

**Table 3:** Association of outdoor ALAN exposure with gestation day.

	β (95%CI)	*p*
Model 1		
T1	−0.151 (−0.217, −0.085)	<0.001
T2	−0.077 (−0.139, −0.015)	0.015
T3	−0.002 (−0.060, 0.056)	0.939
Model 2		
T1	−0.141 (−0.207, −0.075)	<0.001
T2	−0.073 (−0.135, −0.011)	0.021
T3	0.003 (−0.055, 0.061)	0.910
Model 3		
T1	−0.156 (−0.201, −0.111)	<0.001
T2	−0.076 (−0.120, −0.033)	0.001
T3	−0.016 (−0.050, 0.018)	0.368

ALAN, Artificial light at night; T1, First trimester; T2, Second trimester; T3, Third trimester; β represents the partial regression coefficient of the multiple linear regression model. 95%CI, 95% confidence interval; Model 1: Crude model; Model 2: Adjusted for age, ethnicity, gravidity, and parity; Model 3: Further adjusted for normalized difference vegetation index (NDVI), ambient fine particulate matter (PM_2.5_), and ambient inhalable particulate matter (PM_10_), based on Model 2.

### Association of outdoor ALAN exposure in different trimesters with PTB

Table 4 presents the association of outdoor ALAN exposure with PTB using logistic regression models. In crude model, a significant positive association was found between ALAN exposure and PTB in the first trimester (T1: OR = 1.029, 95%CI: 1.023–1.036, *p* < 0.001) and second trimester (T2: OR = 1.018, 95%CI: 1.012–1.024, *p* < 0.001). After adjusting for age, ethnicity, gravidity, and parity, similar results were obtained. In the fully adjusted model, with additional adjustments for NDVI, PM_2.5_, and PM_10_, ALAN exposure remained significantly associated with an increased risk of PTB in the first trimester (T1: OR = 1.032, 95%CI: 1.025–1.040, *p* < 0.001) and second trimester (T2: OR = 1.018, 95%CI: 1.011–1.025, *p* < 0.001), while no significant association was observed in the third trimester.

**Table 4:** Association of outdoor ALAN exposure with PTB.

	OR (95%CI)	*p*
Model 1		
T1	1.029 (1.023, 1.036)	<0.001
T2	1.018 (1.012, 1.024)	<0.001
T3	1.006 (1.000, 1.013)	0.057
Model 2		
T1	1.030 (1.023, 1.036)	<0.001
T2	1.018 (1.012, 1.024)	<0.001
T3	1.006 (0.999, 1.013)	0.073
Model 3		
T1	1.032 (1.025, 1.040)	<0.001
T2	1.018 (1.011, 1.025)	<0.001
T3	1.001 (0.993, 1.009)	0.841

PTB, Preterm birth; ALAN, Artificial light at night; T1, First trimester; T2, Second trimester; T3, Third trimester; OR, Odds ratio; 95%CI, 95% confidence interval. Model 1: Crude logistic regression model; Model 2: Adjusted for age, ethnicity, gravidity, and parity; Model 3: Further adjusted for normalized difference vegetation index (NDVI), ambient fine particulate matter (PM_2.5_), and ambient inhalable particulate matter (PM_10_), based on Model 2.

### Sex difference in the association of outdoor ALAN exposure in different trimesters with PTB

Table 5 presents the sex-specific associations of ALAN exposure with PTB. In the fully adjusted model, after controlling for potential confounding factors, the results indicated no significant difference between males and females (*p* > 0.05). ALAN exposure during the first trimester showed significant associations with PTB in both males (OR 1.033, 95% CI 1.023–1.044, *p* < 0.001) and females (OR 1.032, 95% CI 1.021–1.042, *p* < 0.001). The significant associations persisted for the second trimester in both males (OR 1.019, 95% CI 1.010–1.028, *p* < 0.001) and females (OR 1.017, 95% CI 1.007–1.028, *p* = 0.001). However, no significant association was observed for the third trimester in either males or females.

**Table 5:** Sex-specific associations of ALAN exposure with PTB.

	Male		Female		*p* for interaction
	OR (95%CI)	*p*	OR (95%CI)	*p*	
Model 1					
T1	1.031 (1.022, 1.040)	<0.001	1.028 (1.019, 1.037)	<0.001	0.680
T2	1.019 (1.011, 1.027)	<0.001	1.017 (1.008, 1.026)	<0.001	0.699
T3	1.008 (0.999, 1.016)	0.068	1.004 (0.994, 1.014)	0.442	0.573
Model 2					
T1	1.031 (1.023, 1.040)	<0.001	1.028 (1.019, 1.037)	<0.001	0.625
T2	1.019 (1.011, 1.027)	<0.001	1.016 (1.007, 1.025)	<0.001	0.681
T3	1.008 (0.999, 1.016)	0.081	1.004 (0.994, 1.015)	0.447	0.569
Model 3					
T1	1.033 (1.023, 1.044)	<0.001	1.032 (1.021, 1.042)	<0.001	0.741
T2	1.019 (1.010, 1.028)	<0.001	1.017 (1.007, 1.028)	0.001	0.736
T3	1.002 (0.991, 1.012)	0.700	0.999 (0.986, 1.011)	0.827	0.694

ALAN, Artificial light at night; T1, First trimester; T2, Second trimester; T3, Third trimester; OR, Odds ratio; 95%CI: 95% confidence interval. Model 1: Crude logistic regression model; Model 2: Adjusted for age, ethnicity, gravidity, and parity; Model 3: Further adjusted for normalized difference vegetation index (NDVI), ambient fine particulate matter (PM_2.5_), and ambient inhalable particulate matter (PM_10_), based on Model 2.

## Discussion

To investigate the association between outdoor ALAN exposure and PTB, we conducted a retrospective case–control study. Our study found a significant association between exposure to outdoor ALAN during pregnancy and an increased risk of PTB, as well as a decrease in gestational age after adjusting for confounding factors. Furthermore, the association between outdoor ALAN and the risk of PTB did not differ between male and female infants. Our findings provide evidence supporting the role of outdoor ALAN in the risk of PTB among pregnant women.

In the past few decades, the impact of light pollution on human health has become a global focal point of concern. Numerous studies have explored the association between nighttime light exposure and chronic diseases such as cardiovascular diseases (34), obesity (35), and mental disorders (36). However, there was a relative scarcity of research investigating the association between outdoor ALAN exposure and the risk of PTB during pregnancy. Our study provides the evidence indicating that higher levels of outdoor ALAN exposure during pregnancy were associated with an increased risk of PTB. A prospective cohort study among pregnant women found that higher outdoor ALAN exposure during pregnancy was associated with larger fetal abdominal circumference and a higher risk of macrosomia (37). A study conducted in New Jersey supports our findings, where the authors used visual observations to measure light pollution and identified it as a risk factor for PTB (38). In our study, we utilized remote sensing satellite data to precisely quantify the level of ALAN exposure during pregnancy. Moreover, our investigation went beyond conventional approaches by comprehensively adjusting for crucial environmental variables, such as PM_2.5_, PM_10_, and the NDVI, thereby enhancing the robustness and validity of our research findings. Additionally, the study was conducted in Beijing, a megacity (39), and its results carry significant public health implications for addressing light pollution in densely populated urban areas.

Exploring the window of ALAN exposure associated with PTB in pregnant women was paramount importance for devising targeted intervention measures. The early and mid-stages of pregnancy were critical periods for embryonic and fetal development, as well as being particularly vulnerable to external environmental influences (40). In our study, we found that during the first and second trimester of pregnancy, pregnant women exposed to higher levels of ALAN may experience an increased risk of PTB. This association could be attributed to potential disruptions in pregnant women’s biological clocks and hormonal levels, consequently affecting fetal development (41). However, our research did not reveal a significant association between ALAN exposure during the third trimester of gestation and the risk of PTB. This finding may be attributed to the relative maturity of fetuses during the third trimester of pregnancy, resulting in reduced responsiveness to external environmental factors.

The mechanisms underlying the association between ALAN exposure during pregnancy and the risk of PTB were not yet fully elucidated. Several potential mechanisms may be involved. First, the disruption of the internal circadian rhythm system, responsible for regulating the sleep–wake cycle and physiological processes, could play a role (42). ALAN exposure may disturb the delicate balance of circadian rhythm-related genes, such as CLOCK (43). Disruption of these genes has been linked to altered sleep–wake patterns and reduced melatonin production, potentially impacting fetal neurodevelopment (44). Second, ALAN exposure may influence pregnant women’s hormone levels, particularly melatonin, a hormone critical for regulating circadian rhythms during pregnancy (45). ALAN exposure has been associated with the suppression of melatonin secretion, disrupting the delicate hormonal balance integral to maintaining maternal physiological equilibrium (46). This disruption may extend its influence on fetal development by potentially altering key pathways linked to melatonin’s protective effects on the developing fetus, encompassing neurodevelopmental processes and gestational health (47). Finally, the potential consequences of ALAN exposure on pregnant women extend to the realm of immune and inflammatory responses (48). Additionally, the interactive effects of ALAN with other environmental factors might amplify these immunomodulatory effects (49). In conclusion, the mechanisms linking ALAN exposure during pregnancy to the risk of PTB remain complex and multifaceted, more research was needed to explore these mechanisms.

This study has several limitations. Firstly, in our research, we estimated outdoor ALAN exposure during pregnancy using high-resolution satellite images. However, we did not have data on indoor light exposure and whether participants used blackout curtains during the night, which could potentially lead to exposure misclassification. Future studies should consider collecting information on both indoor and outdoor light exposure. Secondly, while we adjusted for environmental confounders related to PTB, such as environmental particulate matter (21) and greenness (50) at the residential area, we did not account for other potential confounding factors such as noise (51) and socioeconomic status. The lack of this information needs to be addressed and improved in future research. Thirdly, our study adopted a retrospective case–control study design, which limits the ability to establish causality between ALAN exposure and PTB. Therefore, the association between ALAN and PTB needs further confirmation through prospective study designs. Fourth, considering practicality and cost constraints, gestational age was determined based on the last menstrual period, introducing potential bias. Future research endeavors were encouraged to employ ultrasound measurements for a more precise assessment of gestational age. Finally, our study was conducted as a single-center study, with participants residing in the Beijing area and having relatively higher socioeconomic status. Therefore, caution should be exercised when extrapolating the study results to regions with lower economic development. Future research should validate these findings in other regions with different socioeconomic backgrounds.

Despite these limitations, our study still possesses several strengths. Firstly, we elucidated the association between ALAN exposure during pregnancy and PTB, identifying the critical exposure window for this association. This finding provides valuable reference for targeted intervention measures during the identified exposure window. Additionally, we conducted a series of sensitivity analyses and performed stratified analyses by newborn sex to assess the consistency and robustness of this association.

## Conclusion

In conclusion, our study found that higher levels of outdoor ALAN, particularly during first and second trimester, were associated with reduced gestational age and an increased risk of PTB. These findings underscore the potential impact of outdoor ALAN exposure during pregnancy on health and provide new insights into the occurrence of PTB. Our study offers valuable references for policymakers to implement measures to curb the escalating light pollution during nighttime.

## Data availability statement

The raw data supporting the conclusions of this article will be made available by the authors, without undue reservation.

## Ethics statement

The studies involving humans were approved by Institutional Review Board of China-Japan Friendship Hospital. The studies were conducted in accordance with the local legislation and institutional requirements. Written informed consent for participation was not required from the participants or the participants’ legal guardians/next of kin in accordance with the national legislation and institutional requirements.

## Author contributions

QS: Writing – original draft, Writing – review & editing. YY: Investigation, Writing – review & editing. JL: Data curation, Investigation, Methodology, Writing – review & editing. FY: Investigation, Visualization, Writing – review & editing. YC: Data curation, Resources, Writing – review & editing. DL: Software, Writing – review & editing. QZ: Conceptualization, Supervision, Writing – review & editing.

## Footnotes

^1^https://eogdata.mines.edu/

^2^https://ladsweb.modaps.eosdis.nasa.gov

^3^https://map.baidu.com
