# Supplementary material for: Association between exposure to outdoor artificial light at night and the risk of preterm birth
Source: Front Public Health. 2023 Dec 14;11:1280790. doi: 10.3389/fpubh.2023.1280790 (PMC10756648; doi:10.3389/fpubh.2023.1280790)
Supplement: Supplementary file 1 [file Data_Sheet_1.docx]

Association between Exposure to Outdoor Artificial Light at Night and the Risk of Preterm Birth: A Retrospective Case-Control Study


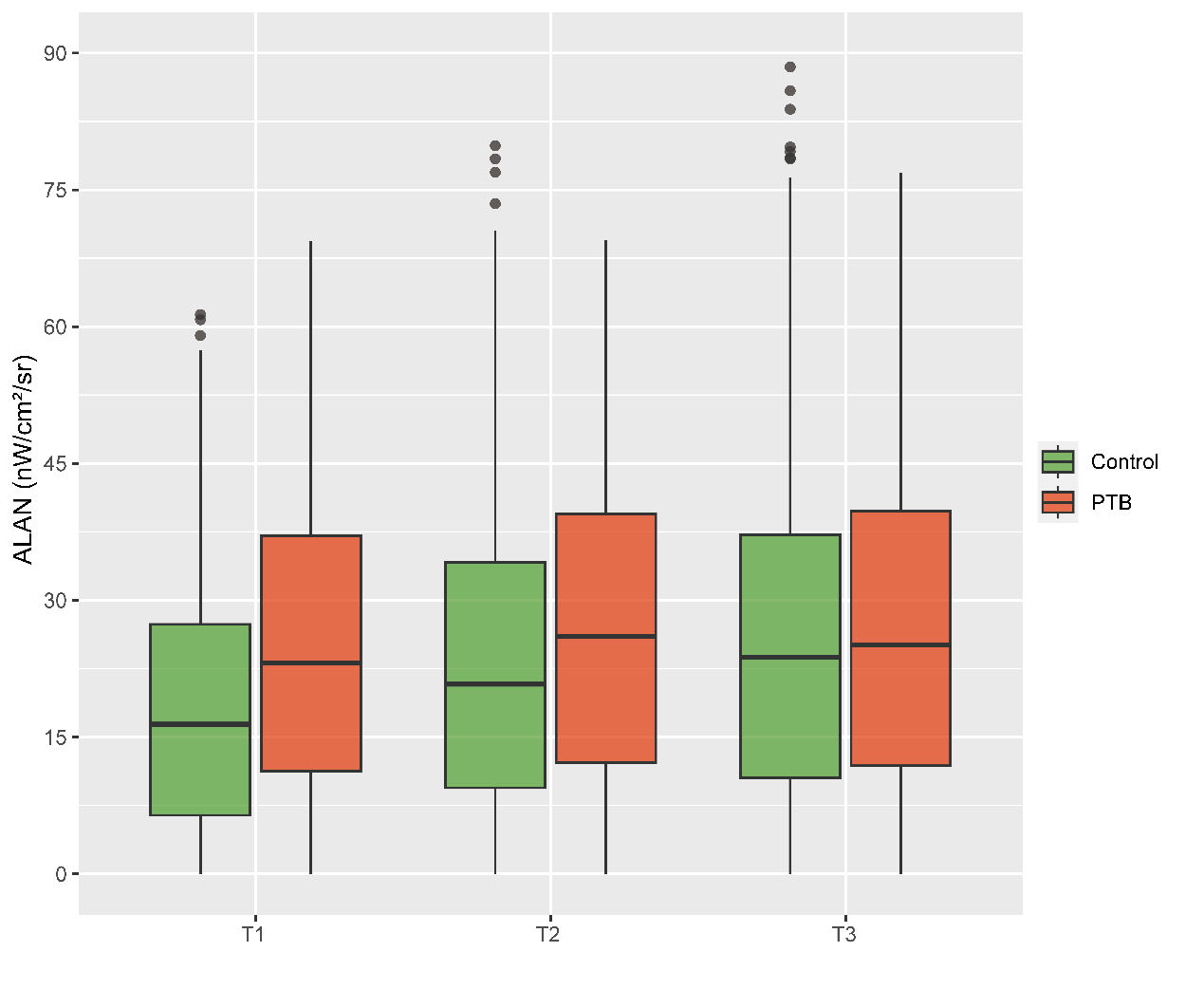


Supplementary Figure S1. Boxplot illustrating differences in outdoor ALAN between the PTB group and the control group.

ALAN: artificial light at night; T1: First trimester; T2: Second trimester T3: Third trimester

Supplementary Table S1. Comparison of ALAN classification between the PTB group and the control group.

| Variables |  | Control group | Case group | *P* |
| --- | --- | --- | --- | --- |
| ALAN _T1_ category (%) | Q1 (≤ 5.10 nW/cm²/sr) | 506 (21.43) | 63 (13.26) | < 0.001 |
|  | Q2 (5.10–11.28 nW/cm²/sr) | 509 (21.56) | 57 (12.00) |  |
|  | Q3 (11.28–19.97 nW/cm²/sr) | 487 (20.63) | 80 (16.84) |  |
|  | Q4 (19.97–32.39 nW/cm²/sr) | 455 (19.27) | 112 (23.58) |  |
|  | Q5 (≥ 32.39 nW/cm²/sr) | 404 (17.11) | 163 (34.32) |  |
| ALAN _T2_ category (%) | Q1 (≤ 7.92 nW/cm²/sr) | 495 (20.85) | 75 (15.79) | < 0.001 |
|  | Q2 (7.92–14.82 nW/cm²/sr) | 498 (20.98) | 73 (15.37) |  |
|  | Q3 (14.82–25.59 nW/cm²/sr) | 485 (20.43) | 83 (17.47) |  |
|  | Q4 (25.59–37.71 nW/cm²/sr) | 461 (19.42) | 109 (22.95) |  |
|  | Q5 (≥ 37.71 nW/cm²/sr) | 435 (18.32) | 135 (28.42) |  |
| ALAN _T3_ category (%) | Q1 (≤ 8.44 nW/cm²/sr) | 477 (20.14) | 63 (19.15) | 0.300 |
|  | Q2 (8.44–18.21 nW/cm²/sr) | 477 (20.14) | 62 (18.84) |  |
|  | Q3 (18.21–30.18 nW/cm²/sr) | 481 (20.30) | 59 (17.93) |  |
|  | Q4 (30.18–40.38 nW/cm²/sr) | 477 (20.14) | 65 (19.76) |  |
|  | Q5 (≥40.38 nW/cm²/sr) | 457 (19.29) | 80 (24.32) |  |

ALAN: Artificial Light at Night; T1: First trimester; T2: Second trimester; T3: Third trimester; Q1-Q5: Categorized into 5 categories based on percentiles.

Supplementary Table S2. Association of gestation day with outdoor ALAN (per 20%)

|  | β (95%CI) | *P* |
| --- | --- | --- |
| Model 1 |  |  |
| T1 | -1.422 (-2.147, -0.698) | <0.001 |
| T2 | -0.811 (-1.536, -0.086) | 0.028 |
| T3 | 0.230 (-0.466, 0.927) | 0.517 |
| Model 2 |  |  |
| T1 | -1.294 (-2.020, -0.567) | <0.001 |
| T2 | -0.772 (-1.499, -0.046) | 0.037 |
| T3 | 0.308 (-0.391, 1.006) | 0.388 |
| Model 3 |  |  |
| T1 | -1.437 (-1.937, -0.937) | <0.001 |
| T2 | -0.766 (-1.280, -0.252) | 0.004 |
| T3 | 0.062 (-0.348, 0.472) | 0.768 |

ALAN: artificial light at night; T1: First trimester; T2: Second trimester T3: Third trimester; β represents the partial regression coefficient of the multiple linear regression model, indicating the change in gestational days per unit increase in ALAN. 95%CI: 95% confidence interval; Model 1: Crude model; Model 2: Adjusted for age, ethnicity, gravidity, and parity; Model 3: Further adjusted for normalized difference vegetation index (NDVI), ambient fine particulate matter (PM_2.5_), and ambient inhalable particulate matter (PM_10_), based on Model 2.

Supplementary Table S3. Association of PTB with outdoor ALAN (per 20%)

|  | OR (95%CI) | *P* |
| --- | --- | --- |
| Model 1 |  |  |
| T1 | 1.398 (1.300, 1.506) | <0.001 |
| T2 | 1.221 (1.138, 1.312) | <0.001 |
| T3 | 1.067 (0.983, 1.158) | 0.120 |
| Model 2 |  |  |
| T1 | 1.402 (1.302, 1.511) | <0.001 |
| T2 | 1.221 (1.137, 1.312) | <0.001 |
| T3 | 1.062 (0.978, 1.153) | 0.153 |
| Model 3 |  |  |
| T1 | 1.414 (1.302, 1.538) | <0.001 |
| T2 | 1.228 (1.132, 1.333) | <0.001 |
| T3 | 0.986 (0.894, 1.089) | 0.786 |

ALAN: artificial light at night; T1: First trimester; T2: Second trimester T3: Third trimester; OR: Odds ratio; 95%CI: 95% confidence interval. Model 1: Crude logistic regression model; Model 2: Adjusted for age, ethnicity, gravidity, and parity; Model 3: Further adjusted for normalized difference vegetation index (NDVI), ambient fine particulate matter (PM_2.5_), and ambient inhalable particulate matter (PM_10_), based on Model 2.

Supplementary Table S4. Sex-specific associations of PTB with outdoor ALAN (per 20%)

|  | Male | | Female | | *P* for interaction |
| --- | --- | --- | --- | --- | --- |
|  | OR (95%CI) | *P* | OR (95%CI) | *P* |  |
| Model 1 |  |  |  |  |  |
| T1 | 1.414 (1.281, 1.563) | <0.001 | 1.380 (1.237, 1.544) | <0.001 | 0.750 |
| T2 | 1.264 (1.149, 1.393) | <0.001 | 1.175 (1.058, 1.308) | 0.003 | 0.320 |
| T3 | 1.078 (0.966, 1.203) | 0.180 | 1.053 (0.931, 1.191) | 0.412 | 0.778 |
| Model 2 |  |  |  |  |  |
| T1 | 1.420 (1.286, 1.572) | <0.001 | 1.381 (1.238, 1.545) | <0.001 | 0.715 |
| T2 | 1.265 (1.149, 1.395) | <0.001 | 1.173 (1.055, 1.306) | 0.003 | 0.309 |
| T3 | 1.072 (0.960, 1.198) | 0.215 | 1.051 (0.929, 1.190) | 0.430 | 0.778 |
| Model 3 |  |  |  |  |  |
| T1 | 1.433 (1.280, 1.608) | <0.001 | 1.394 (1.234, 1.581) | <0.001 | 0.661 |
| T2 | 1.275 (1.142, 1.425) | <0.001 | 1.176 (1.042, 1.329) | 0.009 | 0.290 |
| T3 | 0.983 (0.859, 1.126) | 0.807 | 0.985 (0.852, 1.139) | 0.839 | 0.978 |

ALAN: artificial light at night; T1: First trimester; T2: Second trimester T3: Third trimester; OR: Odds ratio; 95%CI: 95% confidence interval. Model 1: Crude logistic regression model; Model 2: Adjusted for age, ethnicity, gravidity, and parity; Model 3: Further adjusted for normalized difference vegetation index (NDVI), ambient fine particulate matter (PM_2.5_), and ambient inhalable particulate matter (PM_10_), based on Model 2.

Supplementary Table S5. Associations of PTB with outdoor ALAN in Han Chinese

|  | OR (95%CI) | *P* |
| --- | --- | --- |
| T1 | 1.033 (1.026, 1.041) | <0.001 |
| T2 | 1.019 (1.012, 1.027) | <0.001 |
| T3 | 1.002 (0.994, 1.010) | 0.623 |
| T1_20%_ | 1.437 (1.320, 1.567) | <0.001 |
| T2_20%_ | 1.240 (1.141, 1.350) | <0.001 |
| T3_20%_ | 0.998 (0.903, 1.104) | 0.973 |

ALAN: artificial light at night; T1: First trimester; T2: Second trimester T3: Third trimester; OR: Odds ratio; 95%CI: 95% confidence interval. Model 1: Crude logistic regression model; Model 2: Adjusted for age, ethnicity, gravidity, and parity; Model 3: Further adjusted for normalized difference vegetation index (NDVI), ambient fine particulate matter (PM_2.5_), and ambient inhalable particulate matter (PM_10_), based on Model 2. T1: T3_20%_: Every 20% increase in ALAN.

Supplementary Table S6. Associations of PTB with outdoor ALAN in primiparous Women.

|  | OR (95%CI) | *P* |
| --- | --- | --- |
| T1 | 1.036 (1.028, 1.045) | <0.001 |
| T2 | 1.022 (1.014, 1.030) | <0.001 |
| T3 | 1.002 (0.993, 1.011) | 0.652 |
| T1_20%_ | 1.489 (1.351, 1.646) | <0.001 |
| T2_20%_ | 1.271 (1.154, 1.402) | <0.001 |
| T3_20%_ | 0.991 (0.884, 1.111) | 0.873 |

ALAN: artificial light at night; T1: First trimester; T2: Second trimester T3: Third trimester; OR: Odds ratio; 95%CI: 95% confidence interval. Model 1: Crude logistic regression model; Model 2: Adjusted for age, ethnicity, gravidity, and parity; Model 3: Further adjusted for normalized difference vegetation index (NDVI), ambient fine particulate matter (PM_2.5_), and ambient inhalable particulate matter (PM_10_), based on Model 2. T1: T3_20%_: Every 20% increase in ALAN.
